# Micro-to-nanometer patterning of solution-based materials for electronics and optoelectronics

**DOI:** 10.1039/c9ra07514c

**Published:** 2019-11-22

**Authors:** Yo-Han Suh, Dong-Wook Shin, Young Tea Chun

**Affiliations:** Electrical Engineering Division, Department of Engineering, University of Cambridge 9 JJ Thomson Avenue Cambridge CB3 0FA UK ytc24@cam.ac.uk

## Abstract

Technologies for micro-to-nanometer patterns of solution-based materials (SBMs) contribute to a wide range of practical applications in the fields of electronics and optoelectronics. Here, state-of-the-art micro-to-nanometer scale patterning technologies of SBMs are disseminated. The utilisation of patterning for a wide-range of SBMs leads to a high level of control over conventional solution-based film fabrication processes that are not easily accessible for the control and fabrication of ordered micro-to-nanometer patterns. In this review, various patterning procedures of SBMs, including modified photolithography, direct-contact patterning, and inkjet printing, are briefly introduced with several strategies for reducing their pattern size to enhance the electronic and optoelectronic properties of SBMs explained. We then conclude with comments on future research directions in the field.

## Abstract 2

Technologies for micro-to-nanometer patterns of solution-based materials (SBMs) contribute to a wide range of practical applications in the fields of electronics and optoelectronics.

## Graphical abstract

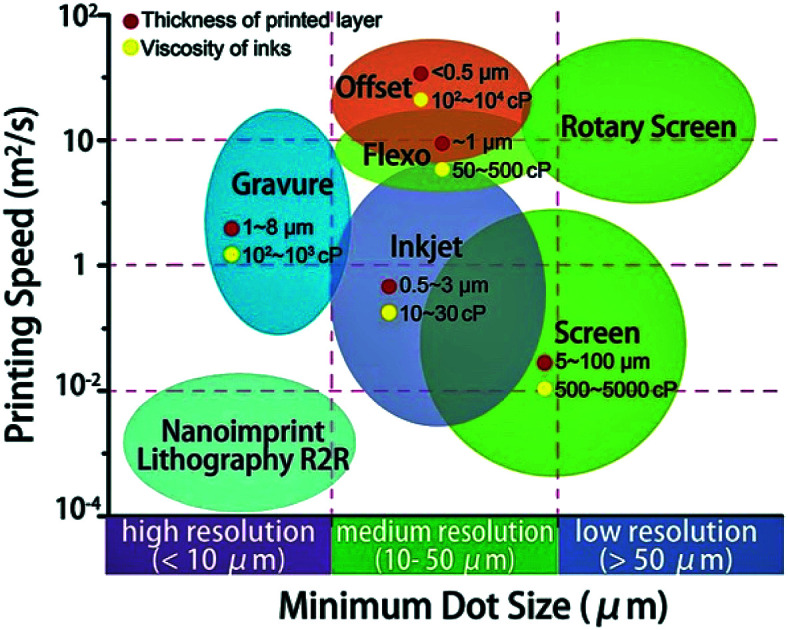

## 1. Introduction

Recently, interest in flexible, stretchable, and wearable electronics has rapidly increased, especially for the displays of information devices.^1–4^ Most flexible substrates consist of polymer materials, which require low-temperature processes for the fabrication of electronics and optoelectronics devices. For example, flexible displays need the integration of circuits with fine pixels onto a flexible substrate. In addition, driving circuits and energy storage devices are required for the operation of flexible information devices. The main challenges to consider for flexible electronics with micro or nanopatterns are low processing temperature and fabrication cost. The patterning processes of solution-based materials (SBMs) are actively researched based on these commercial/industrial demands.^5–7^ Reducing the overall dimension of flexible electronics is necessary for next-generation information devices including microscale energy storage device, energy generation device, wearable electronics, and portable electronics to be integrated with circuits.^2,6,7^

In recent years, the research on micro-to-nanometer scale patterning techniques of SBMs has received increased attention as a strong candidate for the commercialisation in the fields of electronics and optoelectronics. The patterning procedures of SBMs have lower fabrication temperature, fewer process steps, and less waste of materials than conventional patterning procedure including optical lithography, electron-beam lithography,^8^ and scanning probe lithography.^9^ Recently, a sub-10 nm gap structure was reported by using optical lithography with a deep ultraviolet (UV) light source.^10^ However, the UV light source and/or lift-off process could induce degradation of the active layer and scanning probe lithography with a direct-write and mask-less has limited large-area patterning. On the other hand, patterning processes of SBMs are milder and cheaper than conventional procedures and have a broad applicability, benefitting from easily tailoring chemical and physical properties of solutions and substrates. Because of these various possibilities, many researchers are intensively interested in development and optimisation of patterning techniques for SBMs. Here, we introduce state-of-the-art patterning techniques of SBMs to fabricate micro-to-nanometer scale patterns. This work introduces the printing methods,^11,12^ solution ordering from surface treatment,^13^ nano-imprinting,^14,15^ transfer printing,^16,17^ and modified photolithography method^18,19^ to explain the current state of the patterning technology of SBMs. Additionally, various applications, which include various optoelectronics with integrated patterns, are introduced to explain the effects of each patterning procedure.

**Fig. 1:**
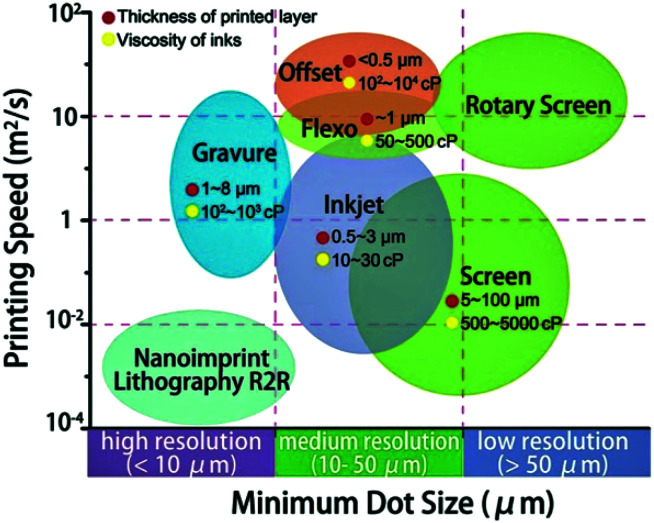
Schematic of various printing technology with printing speed and resolution. These figures have been adapted from ref. 5, with permission from the Royal Society of Chemistry.

We show the schematic for the resolution and printing speed of various patterning technologies (Fig. 1).^5^ Generally, solution ink spreading or film transfers for the desired substrate were used for the patterning of various ink form solutions.^11,20^ The roll-to-roll (R2R) method has a high speed with broad range resolution, the inkjet printing has middle range speed with medium resolution, and the nano-imprint R2R have low speed with high resolution.^5,21^ In addition, photolithography technology is used for patterning with few steps such as light exposure with a photomask, deposition, and lift-off without an etching process. The photoresist mixed solution or photoactive molecule attached quantum dot (QD) is directly applied as a photoresist and photoactive layer.^18,19^ This review introduced a state-of-the-art approaches for micro to nanometer patterning of SBMs that modifies the conventional approach for the solution patterning.

## 2. Nanopatterning of SBMs using optical lithography

Optical lithography (also known as photolithography) techniques that utilise ultra-violet (UV) light sources, such as KrF (248 nm) laser, ArF (198 nm) laser, or even extreme UV (13.5 nm (EUV) or soft X-rays), have been developed for smaller patterning scale (nanometer scale) commonly used in the industry. Thus far, photolithography, which refers to a projection mode, represents the dominant manufacturing approach with physical deposition techniques such as electron-beam (E-beam or thermal) evaporation and sputtering deposition for inorganic electronics and optoelectronics because of its low-cost implementation (mass production), high speed, parallel patterning capability, and high resolution. In academic laboratories, contact photolithography technology commonly utilises a Hg lamp (365, 405, and 436 nm) as a light source to fabricate micrometer-scale patterns of organic and inorganic SBMs because of its low cost, wafer-scale productivity, and accessible applicability to diverse micro-fabrication.^22^

Additionally, in order to define the nanopatterns of resists, modified lithography techniques have been suggested, including near-field photolithography, coupling and guiding light through elastomeric masks,^23–25^ evanescent near-field optical lithography with conformable membrane masks,^26^ employing surface plasmon polariton with periodic metal masks,^27^ and beam pen lithography with metal-coated nanoscale apexes on polydimethylsiloxane (PDMS) masks.^28–30^ In addition, direct writing methods including E-beam lithography (EBL) and scanning probe-based lithography that can provide a high resolution have been suggested to implement fabricating nanopatterns of SBMs into desired substrates. In this chapter we review the materials and processing strategies to form nanopatterned active layer films of inorganic SBMs such as PbS, PbSe, and CdSe cores, and CdSe/CdS core/shell by EBL that appear to have promise for this area.

**Fig. 2:**
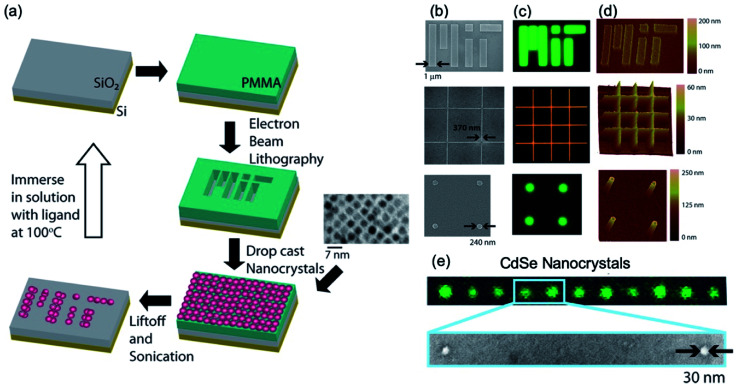
(a) Schematics of the nanoscale patterning processes for semiconductor nanocrystals. The patterns (MIT written), which were formed by EBL on PMMA resist film, are positioned on the surface of the substrate with nanoscale precision. Transmission electron micrographs of the nanocrystal films before and after cap exchange. Nanopatterned films of CdS nanocrystals (first and third rows) and Zn_0.5_Cd_0.5_Se–Zn_0.5_Cd_0.5_S core–shell nanocrystals (second row). The images of (b) SEM, (c) fluorescence, and (d) AFM. (e) The images of green fluorescence and an electron micrograph indicate that the size of the pattern is only about 30 nm. These figures have been adapted from ref. 30, with permission from the American Chemical Society.

Fig. 2 shows the first semiconductor nanocrystal films having nanoscale dimensions that are electrically conductive and crack-free. Mentzel *et al.*^31^ demonstrate the nanoscale patterns with PbS, PbSe, and CdSe cores and Zn_0.5_Cd_0.5_Se–Zn_0.5_Cd_0.5_S core–shell nanocrystals with a variety of ligands.^31^ The patterns have dimensions as small as 30 nm. The details of the procedures to fabricate nanoscale-patterned nanocrystals are as follows (Fig. 2a): silicon dioxide as a substrate is selected because of its prevalence in a variety of device applications, and a 100 nm thick of positive resist, poly(methyl methacrylate) (PMMA), is spin-coated on a substrate approximately 5 mm × 5 mm for EBL. This approach holds for films of various types of nanocrystals such as PbS, PbSe, and CdSe cores and Zn_0.5_Cd_0.5_Se–Zn_0.5_Cd_0.5_S core–shell, which have been shown in the literature^31^ and achieves an arbitrary shape and size as small as 30 nm. Specifically, 30 nm to 1 μm films of nanocrystals patterned with EBL, drop-cast, and a lift-off process in order are shown in Fig. 2b–e, which were observed by fluorescence microscopy, scanning electron microscope (SEM), and atomic force microscope (AFM).

**Fig. 3:**
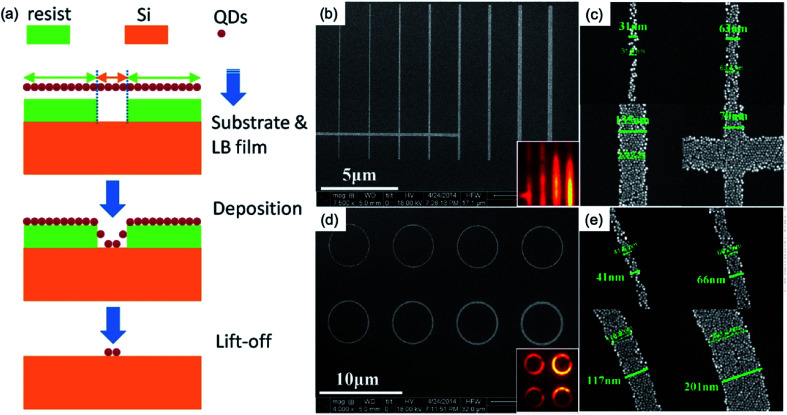
SEM images of monolayered the nanoscale patterns for QD films. (a) Schematics of the experimental flow of the patterning of QDs. (b) Line and (c) ring patterns with different widths, and the enlarged views of (c) and (e) of some selected widths for the line (b) and ring (d) patterns, respectively. These figures have been adapted from ref. 31, with permission from the American Chemical Society.

Nanoscale patterns of QDs have been demonstrated by Xie *et al.*, which is based on EBL and a lift-off process.^32^ Here, in comparison with the drop-casted nanocrystals film of Fig. 2, the Langmuir–Blodgett (LB) deposition technique is adapted to achieve the deposition of monolayer QD films with nanopatterns. They introduce the LB deposition technique combined with EBL^33,34^ by providing excellent control over the pattern structure and maintaining a well-defined QD surface density in the monolayer.^32^Fig. 3a illustrates the proposed processing scheme. First, by using EBL, they defined the desired pattern in a diluted ZEP 520A resist film with an initial thickness of approximately 40 nm. Oleate passivated CdSe/CdS core/shell QDs were synthesized by a seeded growth flash approach^35^ with a diameter of approximately 10 nm and a central emission peak of about 650 nm. Lastly, a lift-off process was performed, and the resist was removed from the substrate, leaving the patterned QDs behind.^32^Fig. 3b–e shows the nanoscale patterns down to 30 nm feature size without any observable re-deposition of free QDs or tearing of patterns. In addition, a 30 nm trench pattern in a 20 nm thick resist film was observed.

**Fig. 4:**
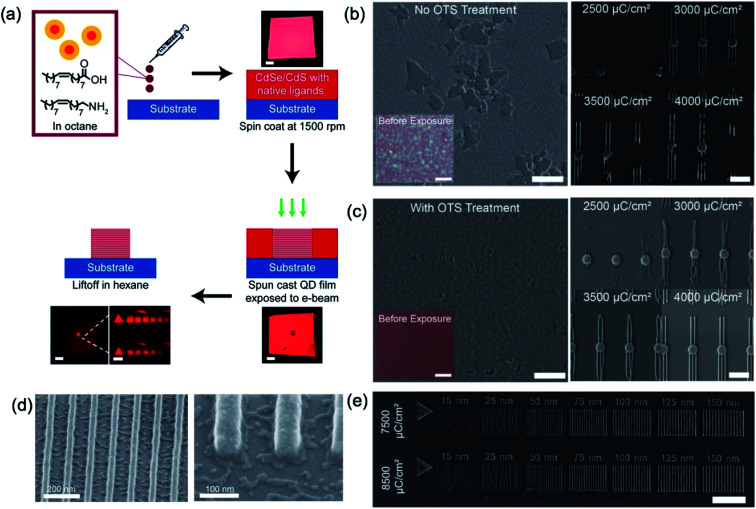
(a) Schematic of basic QD patterning process and photographs of QD-coated substrate during processing. (b) and (c) SEM images of QD film on the substrate before and after OTS treatment and direct patterned QD films by E-beam. Scale bar is 5 (first row) and 2 (second row) μm. (d) SEM images of 30 nm patterned QD film. (e) SEM image of patterned QD lines ranging from 15 to 150 nm thick with the E-beam dose increasing from 7500 to 8500 μC cm^−2^. Scale bar is 3 μm. These figures have been adapted from ref. 17, with permission from the American Chemical Society.

Recently, the direct nanopatterning of QD films using EBL on substrates treated with a self-assembled monolayer (SAM) of octadecyltrichlorosilane (OTS) has been reported without the resist activated by electron beam, which is allowed to create feature sizes as thin as 30 nm with heights of multiple layers and characterise the pattern resolution, robustness, and placement accuracy. Dement *et al.* chose to explore direct EBL patterning of CdSe/CdS core/shell QD thin films because of their high quantum yield and the stability provided by the CdS shell.^18^ Basically, spin-coated QD films are easily re-dispersed by washing with the initial solvent such as hexane or octane. After the irradiation of QD films by electron beam or X-ray,^36^ the solubility of the exposed area to the initial solvent is degraded more than that of as-spun QD film. As a result, the exposed region can no longer be washed away during the lift-off step, as shown in Fig. 4a. In addition, they performed the surface functionalisation of the substrate with OTS to enhance the wettability of QD solution onto both Si and Al_2_O_3_-coated Si substrates (Fig. 4b and c), resulting in better film uniformity, and obtaining substantially improved QD feature resolution (Fig. 4d). A series of 3 μm long lines with widths from 15 to 150 nm were patterned by EBL (5 nm step size and 5 nA beam), as shown in Fig. 4e. With increasing does from 3500 to 8500 μC cm^−2^, more and more defined patterns in narrow widths were observed (Fig. 4e).

**Fig. 5:**
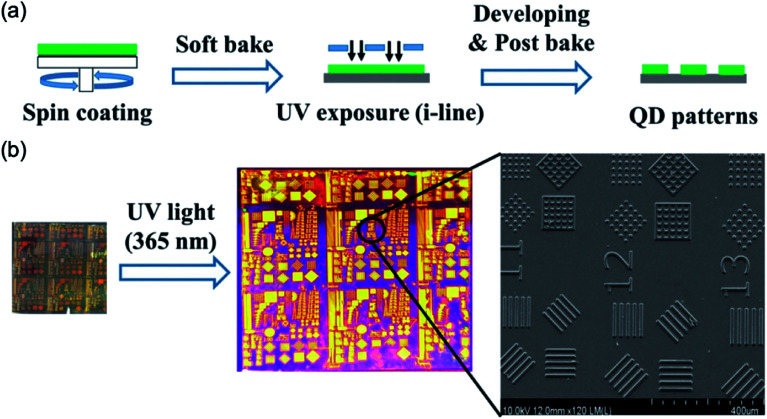
(a) A conventional photolithography procedures such as QDs deposition (spin coating), exposure with a mask, and lift off for patterning of QDs. (b) Optical and SEM images of patterned QDs. These figures have been adapted from ref. 18, with permission from the Wiley-VCH.

Fig. 5 shows microscale patterning of QD from a conventional photolithography procedure with a mixture of QD solution with a photoresist.^19^ This procedure used normal ultraviolet (UV) light sources with pristine ligands without ligand exchanges of QD which were well prevented photoluminescence (PL) degradation of QD material. The oligomer (PO 94F), Irgacure OXe01, and dipropylene glycol diacrylate were used as the photoactive dispersant for CdSe@ZnS QDs. These materials were mixed likely cocktails into propylene glycol monomethyl ether acetate (PGMEA) solvent to create stable colloidal solutions. For the patterning, the conventional photolithography procedures including spin coating, UV exposure, and develop, were exploited as shown in Fig. 5. The surface defect or aggregates on the patterned image were not observed.

**Fig. 6:**
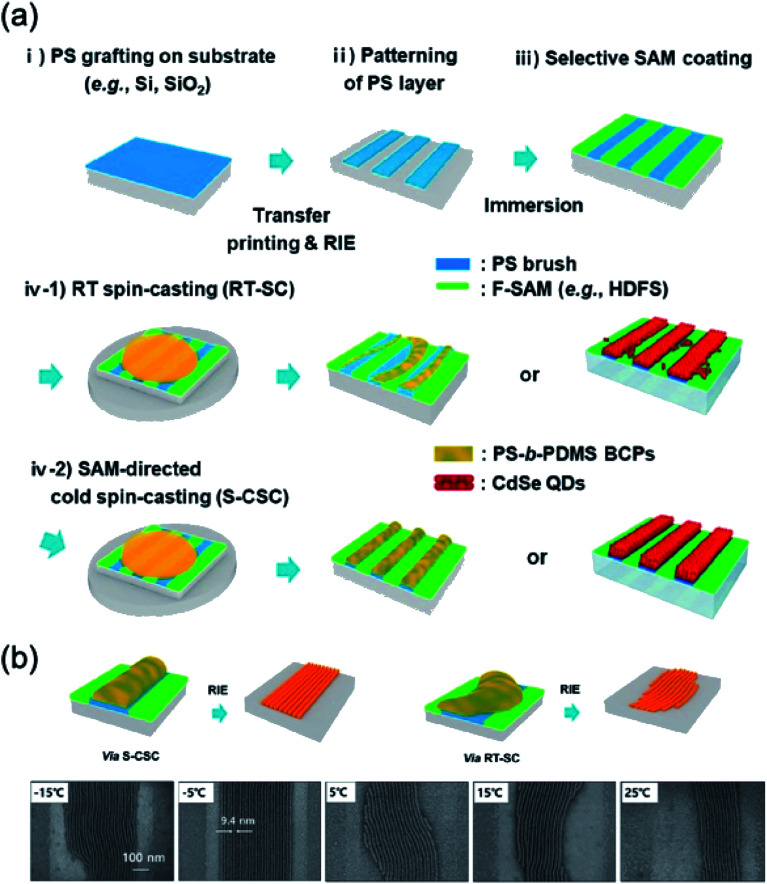
(a) Process flow of SAM-directed cold spin casting. (b) Schematics and SEM images of microdomains of the directed self-assembly of the block-copolymer with the S-CSC procedure. These figures have been adapted from ref. 12, with permission from the American Chemical Society.

Fig. 6 shows the SAM-directed cold spin-casting (S-CSC) procedure. Patterned polystyrene (PS) with a SAM layer was used for making surface tension differences between the PS layer and the SAM layer. The patterned structures were made *via* simple spin casting at the PS with SAM treated substrate. The SAM and patterned PS layer provided directed paths without the bank structure, inducing a surface tension difference. The lower temperature (S-CSC) than the room temperature (RT spin-casting, RT-SC) was applied onto the substrate to increase the uniformity and result in a more aligned pattern due to reducing the evaporation rate of the solvent during the spin-casting procedure, as shown in Fig. 6a. The block-copolymer with S-CSC procedure was used for nanoscale patterning (Fig. 6b). At the −5 °C condition, this paper reported a 9.5 nm sized pattern-to-pattern distance with simple spin casting of the block-copolymer solution. A conventional RT-SC process could make a pattern structure, but the pattern uniformity was low. The S-CSC procedure with a block-copolymer was a uniform micro-pattern form-factor for nanoscale patterning *via* a simple solution-based spin casting procedure.^13^

## 3. Nanopatterning of SBMs using nano-imprint technology (NIT)

The processing technology for making fine patterns must also be developed in accordance with the requirements of the equipment and processes. However, the photolithography technology used in the conventional silicon semiconductor process is limited to the process limit to realise the line width of next-generation devices, and the enormous investment in new equipment must be preceded. Next-generation lithography in this chapter is proposed to fabricate very fine patterns of tens of nanometers.

NIT was introduced as new technology to realise a nanoscale line width. Conventional light-based lithography has problems such as mask material, high energy light source and development of photoresist because of the reduction of light wavelength, but the nano-imprint process does not consider the above problems. It is a simple process, and the equipment itself has the advantage of being much cheaper than the EUV or X-ray lithography equipment.

The nano-imprint process is largely a pattern transfer process through heat treatment and curing by UV irradiation. First, to perform the imprint process, it is necessary to produce a stamp which serves as a mask pattern of the photolithography process. On the mask, the desired pattern is derived from the surface in an embossed pattern. When nano-sized stamps contact a substrate coated with a polymer and heat is applied thereto while applying pressure, the polymer becomes fluid and forms a pattern while filling between the stamp patterns. After cooling, the stamp is removed from the substrate, in order to complete the pattern definition, an additional oxygen plasma dry etching step is used to remove the residual resist layer^37^ that was present between the mold protrusion and the substrate to form a nano/micro scale pattern finally. Because heat is applied, the thermal expansions of the stamp and the substrate must be considered, and there is a high risk that the nano-sized protruding portion of the stamp is broken because the relatively high pressure.

This problem can be complemented by a UV-based imprint process, and most of the imprint processes are currently performed on a UV basis. First, when a low viscosity hardening resist is introduced onto the substrate by coating or dipping, contact with the stamp has the advantage that the fluid type resist can be effectively filled between the stamp patterns even at low pressures. A rigid pattern is then formed while the resist is cured by sensitizing the resist through a transparent stamp with a UV light source. This process is performed at room temperature and has a short curing time, which is advantageous for a quick process. Furthermore, this process can perform pattern alignment through the transparent substrate, which is advantageous for the processing of devices with bottom-up structures.

NIT is used in various applications and is currently undergoing mass production in the memory industry with tens of nanometers. NITs offer various surface nanopatterning possibilities of SBMs and have been developed in the past two decades for various applications including solar cells, light-emitting diodes (LEDs), transistors, and sensors. NITs including micro-contact printing, mould-assisted lithography, hot embossing, and capillary moulding are nonphotolithographic methods that can provide technologically simpler and cheaper nanofabrication strategies, resulting in the potential of high throughput. In 1995, the research team at the University of Minnesota first reported to pattern solution-based organic material directly using nano-imprint lithography technology.^38^ There have been many technological advances since then. However, most of them are indirect patterning using stamped moulding of nanostructure. Therefore, there is still a limit to the patterning of solution-based organic polymers that are vulnerable to UV or high temperature. However, if the direct nano-imprint of the patterning method is developed in the future, the spread effect is expected to be very large.

### 3.1 Embossing

Hot embossing is essentially the stamping technique of a pattern into organic or inorganic SBM films softened by raising the temperature of the polymer just above its glass-transition temperature or sol solvent evaporation temperature, respectively. The stamp used to define the pattern in the organic or inorganic SBM films can be generally made by replication from Si master mould with the nanopatterns. The benefits of this approach are the ability to take advantage of the wide range of properties of organic or inorganic SBM films, as well as the potential to economically mass produce parts with nanometer-scale features. Here, we review the technologies studied previously, which are the techniques of embossing by using a PDMS stamp or Si master mould to make the nanopatterned inorganic and organic SBM films such as TiO_2_, ZnO, PEDOT:PSS, and QDs for electronics and optoelectronics.

**Fig. 7:**
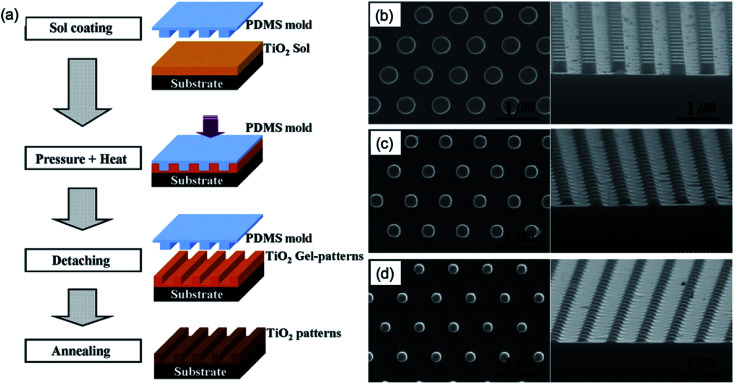
(a) Schematic of the processes of nanopatterned TiO_2_ based on embossing TiO_2_ sol. SEM images of (b) nanopatterns of Si master template, (c) embossed TiO_2_-gel patterns at 200 °C for an hour and (d) nanopatterns of polycrystalline TiO_2_ after annealing at 700 °C in atmospheric ambient for an hour. These figures have been adapted from ref. 13, with permission from Elsevier.

Nanopatterned TiO_2_ with a high surface area has been extensively studied for applications to photocatalysts and photovoltaic devices.^39^ The convenient embossing procedure of a TiO_2_ sol film to obtain nanostructures was demonstrated.^14^ Initially, an ethanol-based TiO_2_ sol was prepared using tetraorthotitanate, diethanolamine and ethanol under stirring as a precursor.^40^ To fabricate the pattern of a nanodot, a PDMS mould with nanodot patterns to which a Si original master template was replicated using a nano-moulding process was used as embossing template. As shown in Fig. 7a, the TiO_2_ sol solution was spin-coated onto an oxidized Si substrate at 7000 rpm for 30 s. Then, the PDMS mould with the nanodot pattern (Fig. 7b) was placed onto the TiO_2_ sol spin-coated substrate and embossed with the pressure of 5 atm and heat at 200 °C for an hour. Afterward, the PDMS mould was removed, and the nanopatterned TiO_2_ gel (Fig. 7c) was annealed at 700 °C for an hour in ambient atmosphere. Lastly, the nanopatterned TiO_2_ film was obtained (Fig. 7d).

**Fig. 8:**
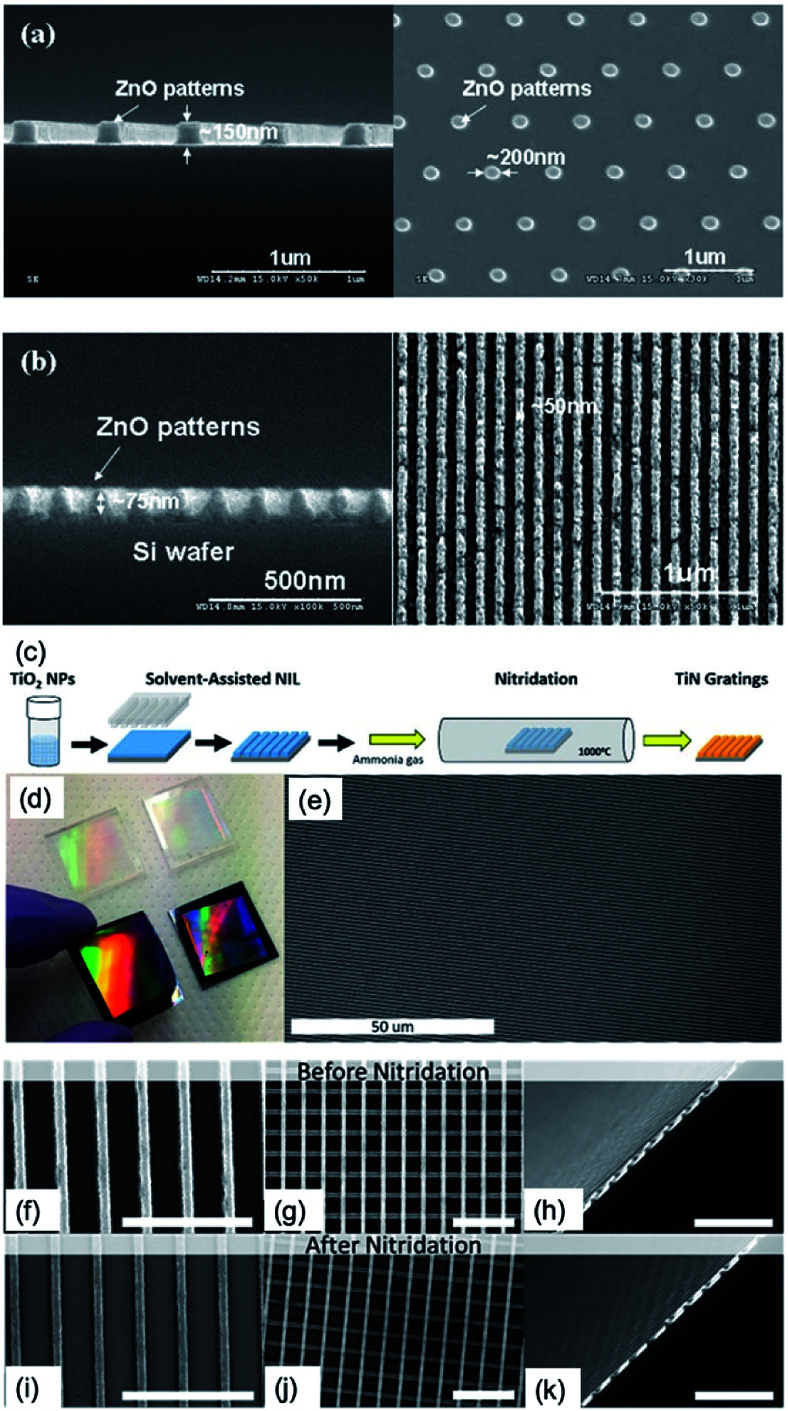
SEM images of ZnO patterns using an h-PDMs mould on the Si substrate. (a) ZnO dot nanopatterns using h-PDMS mould, derived from the Si master mould. (b) 50 nm sized ZnO line nanopatterns on Si substrate (these figures have been adapted from ref. 44, with permission from the Elsevier). (c) Fabrication method for patterned TiO_2_ and TiN film. (d) Centimeter-scale PDMS stamps and embossed gratings on silicon. (e) Large-area SEM image of uniform, defect-free, embossed gratings. SEM images before (f–h) and after (i–k) 6 hours nitridation treatment. All scale bars are 3 μm (these figures have been adapted from ref. 46, with permission from IOP Publishing).

In addition, nanopatterned ZnO and TiN based on embossing technique have been demonstrated. ZnO is an attractive oxide semiconductor with a wide band gap (3.3 eV)^41^ and large excitonic binding energy (60 mV)^42^ that enables application to various fields such as thin-film gas sensors,^43^ photo-detectors,^44^ and LEDs,^45^ especially for the UV region. ZnO sol was prepared by dissolving zinc acetate 2-hydrate in DMF with diethanolamine (DEA),^46^ and the embossing procedure was similar to that of Fig. 7: the ZnO sol film spin-coated at 2000 to 4000 rpm for 60 s is embossed by the h-PDMS mould, which was replicated by the Si master pattern under a pressure of 500 kPa at 200 °C. Afterward, nanopatterned ZnO gel was obtained, and it was annealed at 650 °C for 1 h in ambient atmosphere using rapid thermal annealing, resulting in the formation of crystalline ZnO. Lastly, approximately 200 nm of nanodot patterned ZnO as well as 50 nm of line nanopattern of ZnO can be obtained by the proposed embossing technique, as shown in Fig. 8a and b.

TiN has been proposed as an alternative plasmonic material because of its very high melting point, chemical stability, tunable dielectric function, and fabrication methods compatible with existing manufacturing techniques.^47^ Among various techniques to fabricate TiN films, a scalable, solvent-assisted soft NIT (embossing) method to quickly generate large areas of nanopatterned crystalline TiO_2_ or TiN surfaces and structures has been demonstrated.^48^Fig. 8c shows the fabrication procedures of nanopatterned TiN films. The TiO_2_ nanoparticles in 1,2-propanediol (purchased from US Research Nanomaterials, Inc.) were spin-coated onto the substrate at 3000 rpm for 15 s. The PDMS mould was placed on the still-wet TiO_2_ film and then heated at 50 °C for 5 min. Lastly, with removing the PDMS mould, the nanopatterned TiO_2_ was left on the substrate (Fig. 8d and e). To convert TiO_2_ to TiN, nanopatterned TiO_2_ films are placed in a vacuum chamber at 1000 °C with an ammonia gas flow of 200 sccm for over 4 hours (Fig. 8f–k).

**Fig. 9:**
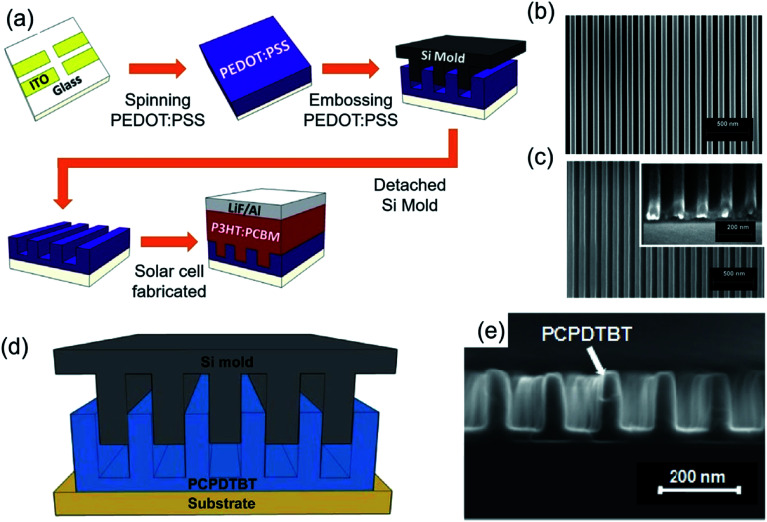
(a) Schematic of ITO patterned, PEDOT:PSS spin-coated, PEDOT:PSS embossed by Si master mould (formation of nanogratings), and P3HT:PCBM spin-coated and thermal evaporation of LiF and Al. SEM images of (b) Si master mould with nanograting pattern and (c) embossed PEDOT:PSS with dehydration (these figures have been adapted from ref. 50, with permission from IOP Publishing). Process flow to form ordered PCPDTBT/C_70_ heterojunction: (d) schematic and (e) SEM image of embossed PCPDTBT (nanogratings) (these figures have been adapted from ref. 14, with permission from the American Chemical Society).

Research progress in organic photovoltaic (OPV) devices has advanced tremendously, driven by the potential for low cost, large area and flexible devices.^49,50^ The power conversion efficiency (PCE) of OPV devices needs to be improved, because its PCE is lower than other types of inorganic solar cells. To achieve this goal, one emerging method, NIL, has been suggested which provides an ordered and continuously interdigitated morphology in active layers for both efficient charge separation and collection.^51^ PEDOT:PSS that is usually used to fabricate solar cells and light-emitting devices as hole transfer layers (HTLs)/electron blocking layers because of their band structure that is compatible with the active layer is spin-coated and embossed by a Si master mould with a 70 nm nanograting pattern at 100 °C and 2 MPa pressure. Then, P3HT:PCBM and LiF and Al were deposited by spin-coating and thermal evaporation, respectively, to fabricate the OPV devices (Fig. 9a). Well-defined PEDOT:PSS with a nanograting pattern (height 60 nm and width 70 nm, Fig. 9c) as well as the mould of Si master pattern is observed by SEM (Fig. 9b). As a result, PEDOT:PSS with the nanograting pattern has higher PCE than that of non-embossed PEDOT:PSS (flat) because of the enhanced hole collection efficiency.^52^ In addition, the embossing technique can be applied to low bandgap polymer solar cells because the active layer has well-ordered heterojunction. The high-quality low bandgap conjugated polymer poly[2,6-(4,4-bis(2-ethylhexyl)-4*H*-cyclopenta[2,1-*b*;3,4-*b*′]-dithiophene)-*alt*-4,7-(2,1,3-benzothiadiazole)] (PCPDTBT) was spin-coated and embossed by the Si mater mould at 170 °C and 5 MPa pressure (Fig. 9d). The nanograting size of embossed PCPDTBT in the width and pitch was varied from 280 nm to 60 nm and from 280 nm to 80 nm, respectively (Fig. 9e). The cell structure was ITO/PEDOT:PSS/PCPDTBT/C_70_/Al, and PCPDTBT with the nanograting with a width of 60 nm width and a pitch of 80 nm has the highest PCE, about 5.5%.^15^

**Fig. 10:**
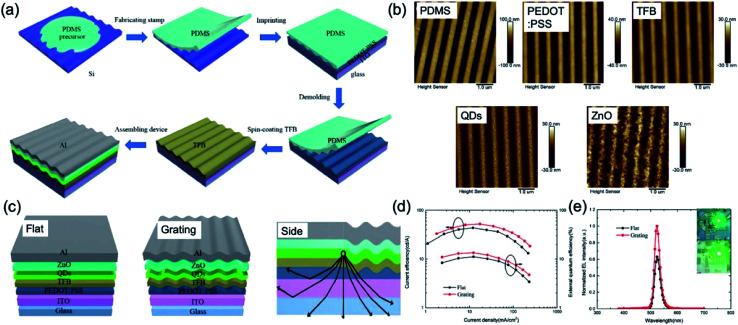
(a) Schematic fabrication process of a QD light-emitting diode (QLED) device with grating nanostructures. (b) AFM images of the surface of PDMS mould, PEDOT:PSS, TFB, QDs, and ZnO. (c) Schematic images of the flat and grating device structures of QLEDs and explanation of the mechanism for improving device out-coupling efficiency with grating nanostructures. (d) Current efficiency and external quantum efficiency as a function of current density. (e) Normalised electroluminescence (EL) spectra. The insets are photographs of the fabricated QLED with and without grating nanostructures. These figures have been adapted from ref. 53, with permission from the Royal Society of Chemistry.

Recently, LEDs based on QDs have received intense interest in the past two decades because of their excellent characteristics such as high brightness, narrow emission bandwidth, high stability and easily tunable emission wavelength by changing the size and composition of the QD.^53,54^ Above all, internal and external light extraction methods from the device have highly demanded in QD-LED (QLED) to increase the limited optical out-coupling efficiency of device.^55^ One of the solutions is the NIL technique. The PEDOT:PSS layer as the HTL was spin-coated on the pre-cleaned ITO glass substrate and embossed using the grating-structured PDMS mould at 80 °C and 10 bar pressure for 300 s. Afterward, PEDOT:PSS with nanogratings was annealed, and other layers for QLED were deposited in order (Fig. 10a). The detailed surface morphology of each layer deposited in order were characterised by AFM (Fig. 10b). The heights of the PDMS, PEDOT:PSS, TFB, QDs, and ZnO nanostructures are about 116.1 nm, 40.5 nm, 27.3 nm, 21.8 nm and 19.6 nm, respectively. Fig. 10c shows the mechanism for enhancing light extraction with a grating functional layer. Basically, the light out-coupling efficiency of the QLED has been assumed to be about 20% because of the critical angle of total internal reflection. Traditionally, the light outcoupling efficiency (extraction efficiency) of device leads to a great loss of light emitted from QDs layer toward the glass substrate, due to total internal reflection into substrate and waveguiding modes and self-absorption of ITO/organic layers, such as PEDOT:PSS and TFB. In the device of flat and grating, the EQE of the device with nanograting increases from 11.13% to 13.45%, and the luminous efficiency increased from 29 010 cd m^−2^ to 44 150 cd m^−2^.

### 3.2 Transfer printing

**Fig. 11:**
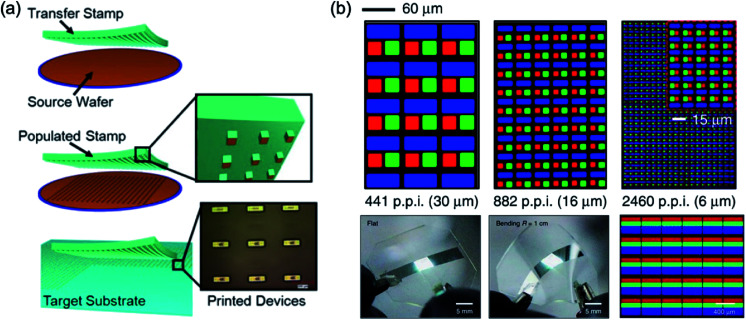
(a) Schematic illustration of the transfer printing procedure, and (b) microscale pixel fabrication by transfer printer with an intaglio stamp. These figures have been adapted from ref. 3, with permission from the Wiley-VCH and ref. 17, with permission from the Nature Publishing Group.

Fig. 11a shows the overall procedure of the transfer printing method. The meaning of the transfer printing is that the pre-deposited film at the donor substrate was transferred to the desired substrate. The patterned PDMS stamp was used as a master stamp for the selective detaching of the film at the donor substrate.^3^ Then, the pattern shape and dimension were well controlled *via* shape control of PDMS stamp or intaglio patterned acceptor substrate.^16,17^

For the commercialisation of the QD display, the active-matrix QD display was reported *via* Kim *et al.*^16^ The patterned PDMS stamp was used for the pixelation of the red, green, and blue QD *via* transfer printing. Afterward, the intaglio transfer printing was reported by Choi *et al.*^17^ Generally, the PDMS stamp had problems reducing the pattern dimension on the stamp below tens of micrometers because its softness. The intaglio stamp with nanopatterns on the hard substrate was allowed to well solve the limitation of the essential problem of intrinsic characteristics of the PDMS. This paper reported 2460 pixels-per-inch (ppi) pixel resolution, which the pixel dimension was 6 μm scale, as shown in Fig. 11b.^17^ In addition, the white QLED with pixilation of the red, green, and blue QD was demonstrated.

**Fig. 12:**
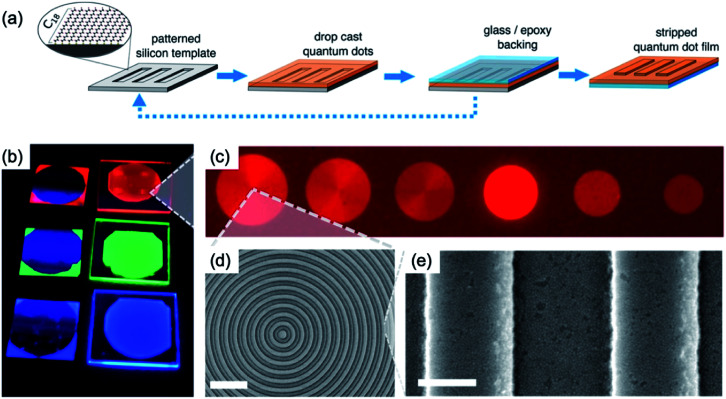
(a) Schematic of fabrication method of QDs nanoscale pattern using patterned silicon template by stripping. (b) Photo-image and (c) fluorescence image of patterned QD film under an ultraviolet light source. (d) SEM image of the patterned QDs film. (e) The enlarged view of (d). Scale bars of (d) and (e) are 2 μm and 200 nm, respectively. These figures have been adapted from ref. 56, with permission from the American Chemical Society.

We show the reverse transfer printing using SAM treated silicon stamp with patterns to obtain the nanoscale patterns of QDs film, as shown in Fig. 12.^56^ Generally, the conventional transfer printing consists of the flat SAM treated silicon substrate used as the donor substrate and the patterned PDMS used for selective detaching of film. In the case of the striping method of patterned QDs (reverse transfer printing), the QDs deposited on a patterned silicon template utilised as a donor substrate, and glass/epoxy backing was used for the selective detaching of the QD layer (Fig. 12a). Fig. 12b and c shows photo-image and fluorescence image of QDs film with a bull's-eye pattern on a glass/epoxy under an ultraviolet light source. The enlarged view of QDs film was observed by SEM, with 550 nm of the pitch size and 300 nm of the pattern size (Fig. 12d and e). The striping method of SBMs using a patterned silicon template can provide low-threshold, single-mode, and flexible lasing sources for future projection and display technologies.^57^

### 3.3 Direct nanoscale patterning of SBMs

In organic device research, including the development of medical science, optics and electronics, the capability to pattern functional polymers in a specific length and width is critical.^58–65^ The interest and ability of solution-based functional polymer direct nanoscale patterning has started from a wide range of polymer functionalities and pattern applications.^66–71^ The functional polymer is a basic component of a variety of advanced electronic devices. Solution-based organics are materials suitable for these applications because they facilitate large-scale, low-cost fabrication of devices with high performance, and patterning of these materials with well-defined geometric features is required to develop practical devices.^72–74^

For decades, the researchers' focus has been on applying organic semiconducting polymer materials to electronics because they are the only material capable of realizing the development of low cost, large area, flexible and lightweight optoelectronic devices.^75–82^ Several direct patterning studies show that it is possible to produce organic semiconducting materials with better performance using specific patterns rather than classic spin coating.

This research field originated from a unique laboratory that first observed electronic conductivity from polymers that were considered insulated.^83^ Over the last century, we have had many exciting breakthroughs on fundamental and inherent limitations on electrical properties such as carrier mobility.^84–89^ Most of the state-of-the-art electronics based on organic materials have several advantages over inorganic materials, and organic semiconductors are a fundamental component of circuitry in devices.^90–95^ However, such solution-based organic semiconductor materials cannot be patterned by a conventional photolithography method because they lose their characteristics by photoresists or another buffer material used during a lithography process. Thus, many researchers have been working hard to improve the performance of patterning by using direct patterning methods.^96–103^ This chapter focuses on recent developments in bottom-up direct nanoscale patterning of solution-based organic semiconductor and other functional polymers.

### 3.4 Direct patterns with stamp mould

Direct patterning technology is the most efficient way to manufacture organic electronics of structures because of the solution-based polymer materials without additional patterning processes. Directly patterning is that the functional polymer materials are directly patterned onto the wafer or glass without a conventional photolithography processes: *e.g.* various printing methods. However, technologies for directly patterning of SBMs are lagging behind because of main issues they have: diffusing the liquid ink, low resolution of dozens of microscales, difficulty in implementing a three-dimensional structure, and residues left in an undesired place on the substrate. Moreover, obtaining a well-defined organic semiconductor pattern with clarity while having uniformity and crystallinity in a solution-based polymer is highly demanding but challenging. In order to realise the fine nanoscale structure, researchers will make a breakthrough in the field of directly patterning of the solution-based polymer materials. Here, we review various methods that have been developed to directly obtain fine nanopatterned functional polymer materials from patterned stamp moulds.

**Fig. 13:**
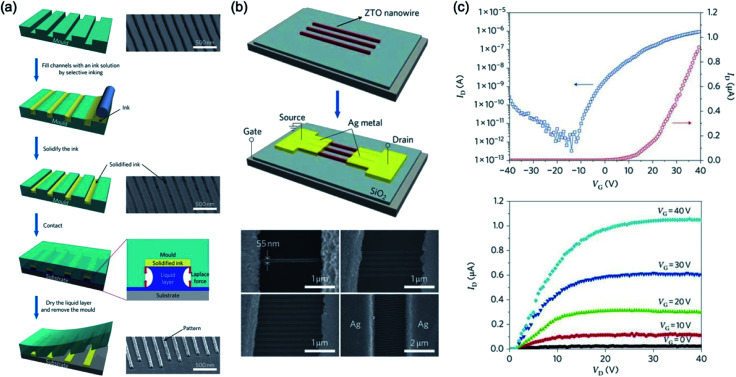
(a) Schematic illustration of LB-nTM and SEM image of the PUA mould and the mould filled with ZTO ink. (b) Schematic diagram of the procedure for fabricating ZTO nanowire FETs using LB-nTM and SEM images of ZTO nanowire FETs. (c) Device performance of the ZTO nanowire FET. These figures have been adapted from ref. 104, with permission from the Nature Publishing Group.

Hwang *et al.*^104^ reported liquid-bridge-mediated transfer moulding (LB-nTM) technology that can perform three-dimensional patterning with nanoscale structures while solving these problems. They claimed that the method that is applied to a wide range of materials creates a variety of functional structures using a wide variety of inks; unlike other direct patterning methods that use liquid inks such as inkjet printing. They can also be used to make nanometer-sized structures without leaving residues in uncoated substrate areas, and because the filled ink is transferred in the coagulated state before being transferred onto the actual substrate, side-diffusion phenomena do not occur. By using the technology, they demonstrated ZTO nanowire transistors had a field-effect mobility of 0.4 cm^2^ V^−1^ s^−1^, on/off ratio of ∼10^6^ and a threshold voltage of 5 V, as shown in Fig. 13.

**Fig. 14:**
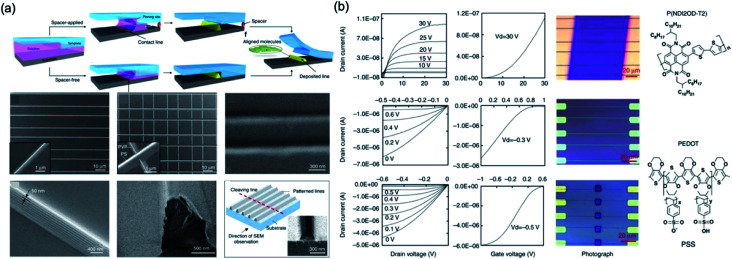
(a) Schematic illustration of the pattern-formation process. The solution is patterned and pinned by the groove corners during drying. (b) Performances and images of devices and molecule structures. These figures have been adapted from ref. 105, with permission from the Nature Publishing Group.

A pinning of capillary bridge technology was investigated by Li *et al.*,^105^ which is that a solution-based polymer is dried on a flexible/rigid substrate under a suspended flexible PDMS template with groove surface and then a nanoscale wire formation fabricated. This group demonstrates an approach with different drying mechanism to fabricate high-resolution structures *via* solvent evaporation in confined geometries. A surface-structured flexible template is used to pattern the liquid into capillary bridges and further guide the liquid-drying process with liquid bridge pinning. This technique is combined with an approach with various drying mechanisms for producing nanoscale structures through solvent evaporation in defined spaces. The PDMS template is used to complete the liquid-drying process with pinning while patterning the solution-based polymer into the capillary bridge effect. They demonstrated N/P-type organic semiconductor nanowire transistors had a field-effect mobility of 0.34 cm^2^ V^−1^ s^−1^ (field-effect mobility ∼0.054 cm^2^ V^−1^ s^−1^ at spin-coating devices with the same material), as shown in Fig. 14.

**Fig. 15:**
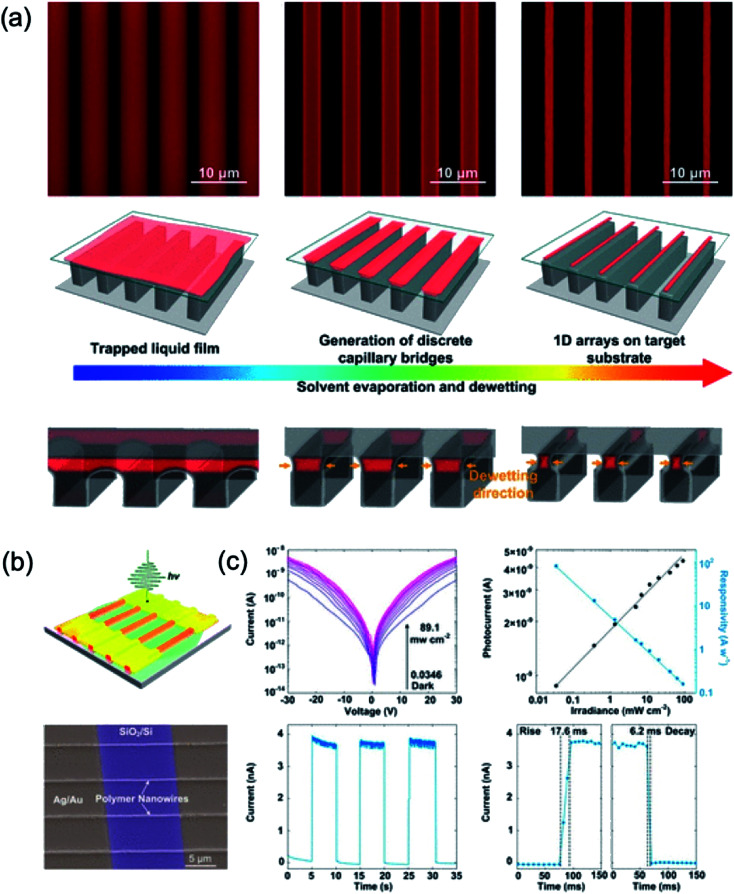
(a) Fabrication of polymer nanowires by controlling dewetting on a template with asymmetric wettability. (b) Schematic and SEM image of photodetectors of polymer nanowire arrays (c) *I*–*V* curves of polymer photodetectors under dark and light illumination with different irradiances and irradiance-dependent photocurrent and responsivity of a device. These figures have been adapted from ref. 106, with permission from the American Chemical Society.

Wei *et al.*^106^ reported using a capillary bridge lithography system to align the polymer nanowires including the molecular packing at the desired location. Applying polymer solutions between them using a template having properties of asymmetric hydrophilic top and hydrophobic sidewalls induce constant packing of the polymer chains through solvent evaporation. The average value of the 100 nanowires generated was about 248 nm in width and 150 nm in height. They demonstrated a high sensitivity of 84.7 A W^−1^ at a wavelength of 532 nm, which is much higher than the spin-coated thin-film photodetector using organic nanowires, as shown in Fig. 15.

### 3.5 Self-assemble direct patterns

While chemical synthesis of soluble functional polymers has reached a highly refined level of specialisation, the reality is that patterning on specific substrates and topographies for organic electronics applications still remains a challenge. To acquire fully nanostructured functional polymers, control of patterning processes is required because of the effect of the chemical structure. In addition, traditional processes such as spin casting often do not fully control the formation of the alignment molecular architecture. Functional polymers with particularly large aromatic moieties are of great interest because they induce molecular self-assembly on a substrate surface. Materials with such localised structures are considered important because they can be useful in functional polymer electronics applications. The nanoscale self-assemble pattern method is still limited, but many studies are underway to devise a new method.

**Fig. 16:**
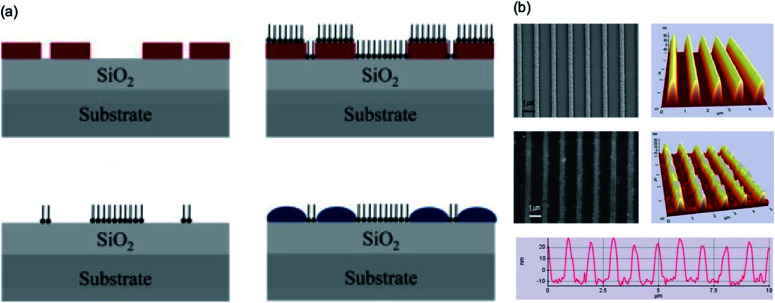
(a) Schematic of conducting polymer patterning using selective surface modification. (b) FE-SEM and AFM images of the nanoscale polymer line patterns with a width of 292 nm at 1 μm spacing produced by using the polymer template. These figures have been adapted from ref. 107, with permission from Elsevier.

Lee *et al.*^107^ developed a hybrid process that combines conventional photolithography and local surface energy modification through hydrophobic treatment in the vapor phase with tridecafluoro-1,1,2,2-tetrahydrooctyltrichlorosilane using a condensation reaction. Fig. 16 shows the selective surface energy modification process and SEM and AFM images of the nanoscale photoresistor patterns using an interference lithography technique. A chemically patterned substrate with locally different surface energies was generated and used to fabricate organic semiconductor field-effect transistors. The polymer solution effectively wetted the selective hydrophobic treatment in the vapor phase with nanoscale hydrophilic regions predetermined by holographic lithography.

**Fig. 17:**
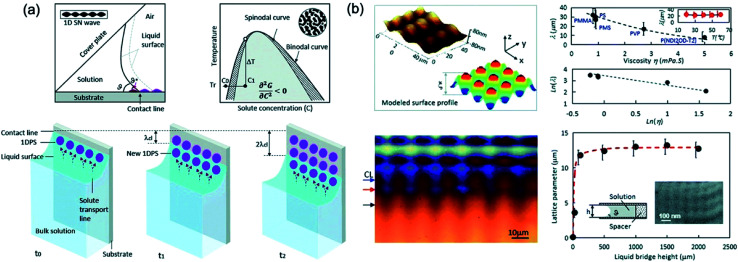
(a) Schematic of Principle of the 2D-array generation from solution and Pinning–depinning of a contact line between the substrate and the solution constrained in a wedge-shaped space. (b) Pattern analysis and formation mechanism. AFM image taken from the PMS pattern and surface profile model and variation in lattice parameters with solution viscosity in different systems, and the temperature dependence of the lattice parameters for the PMS pattern from toluene solution and optical image of a quenched growth front of a 2D array of the PMS polymer during growth from its toluene solution. These figures have been adapted from ref. 108, with permission from the Royal Society of Chemistry.

Li *et al.*^108^ demonstrated the pattern-formation process that can produce 2D arrays of sub-micrometric size through the principle that is similar to optical interference lithography, which is based on the interference between two beams, split from a coherent laser source, forms a standing wave that is recorded on a photoresist coated wafer to fabricate nanoscale pattern.^109,110^ However, the solution based pattern-formation process using two waves are formed through the adjustment of the concentration in a dry solution next to the three-phase line or contact line. One wave arises from the solvent evaporation induced solute condensation next to the contact line with the wave vector next to the contact line. Another wave involved here comes from spinodal-precipitation induced by supercooling consisting of wave vectors along the contact line. Fig. 17 shows the schematic of the principle of the 2D-array generation from the solution and the pattern analysis and formation mechanism. Interference between spinodal waves and solute condensation waves in the contact line allows the formation of a 2D pattern directly from solution with adjustable lattice parameters and lattice type.

## 4. Inkjet printing for micro- to nanoscale patterning

Inkjet printing procedures were well-matured techniques for various patterning and printing applications, which were utilised for the simple printing at paper to micro-circuit fabrication. This method has a simple process with less material waste from direct jetting of the solution for pattern fabrication on the substrate. From these properties, many researchers and companies have focused on the development of inkjet technology.

Inkjet printing methods are composed of the piezoelectric based mechanical jetting and the electrohydrodynamic jetting.^11,20,111–114^ Piezoelectric inkjet printer had piezo-device attached nozzle for fine control of the jetting properties. The volume of the ink was well controlled for the 10 pL scale from the piezo jetting process. The microscale patterns were realised from the pico-liter scale droplets. The electrohydrodynamic jet printing device included a microscale print head with a substrate plate and has an electric potential difference between the print head and the substrate, inducing the jetting of the ink. Even though both printing methods have a common point that is a direct solution jetting onto the substrate, a force to jet the ink is different: the inkjet printing used mechanical force for jetting of the ink and the electrohydrodynamic printing used an electrical field for ejecting the solution onto the desired substrate.

**Fig. 18:**
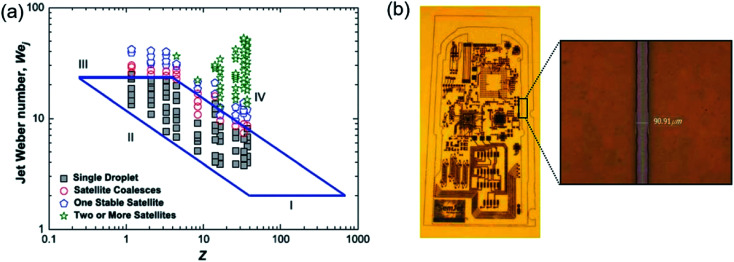
(a) Phase diagram for printability in a parameter space of *Z* and the jet Weber number. (b) Conductive copper pattern inkjetted onto polyimide substrate. These figures have been adapted from ref. 116, with permission from the AIP publishing and ref. 117, with permission from the IOP publishing.

Physical properties of ink formulation are the most important part of the inkjet research field. The viscosity, surface tension, and density of ink solution have to consider during ink formation. All of these factors are directly connected with Reynolds number (NRe), Weber number (NWe), and Ohnesorge number (Oh), which are factors used to characterize properties of a liquid droplet (*Z*) of ink.^115^ Because of the low values of *Z*, the viscous nature of fluid does not allow drop ejection from the nozzle, whereas a higher value of *Z* helps in the formation of a large number of satellite droplets. Stable droplet in the case of drop-on-demand (DOD), which related to rheological properties of ink, is determined by the value of *Z*. Liu *et al.* has found the limiting value of NWe is 2 < NWe < 25, and the limiting value of *Z* is 2 < *Z* < 20 range.^116^ The lower limit of NWe (value = 2) could not generate ink-droplets due to lack of capillary force and the higher limit of NWe (value = 25) leads to the instability of tail in the droplet during drop ejection. Fig. 18a depicts the relationship between NWe and *Z* phase diagram.

With increasing demands for more economic routes to the manufacture of electronic devices, incorporating metal nanoparticle-based printed circuit boards are actively researched. The size of the metal particle in ink has to be considered for stable inkjet processing. Larger particle size and its possibility for aggregation in ink enable to cause clogging of the nozzle. To obtain the high resolution of patterns, the technology to develop ink with smaller metal particle size is required.^115^ Copper nanoparticles (∼30 nm) ink allowed conductive copper pattern with 90 μm using inkjet processing, as shown in Fig. 18b.^113^ The dispersion stability of the nanoparticle ink was treated as a key factor with respect to inkjet printing performance. The stabilizer of the smaller metal nanoparticle size was brought to increasing dispersion stability and maintaining a significant amount of copper nanoparticle without aggregation of the metal nanoparticle.^115,117^

**Fig. 19:**
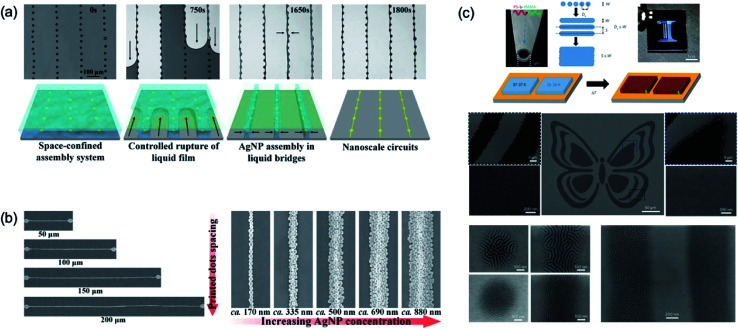
(a) Schematic and SEM image for fabrication of nanoscale circuits using space-confined assembly inkjet printing method. (b) SEM images of well controlled line width of nanoscale circuits by changing concentrations of the Ag nanoparticle solution. (c) Schematic illustration of the process for high-resolution jet printing by the action of electrohydrodynamic forces with block-copolymer. These figures have been adapted from ref. 11 and 107, with permission from the Nature Publishing Group.

Fig. 19a shows nanoscale patterning with piezo-type inkjet printing. The dot pattern was fabricated from inkjet printing and the space-confined assembly method used for a dot-to-dot connection. The single droplet of ink of the piezo-type inkjet printer has a micro-size domain,^11^ which induced difficulties to implement of nano-sized patterns with inkjet process. For nano-scale patterning *via* the inkjet printing process, the space-confined assembly method with simple wettability control of the substrate was introduced. First, ink droplets with hydrophilic solvent were printed on a hydrophobic substrate. After printing the droplets, ink droplets were dewetted into dome-shaped dots. The ink solution was dropped onto the patterned substrate and covered with a flat plate, which the step is called a space-confined assembly system. The nanoscale circuits were demonstrated from SEM image of dot patterning with a space-confined assembly. In addition, the line width of the nanoscale circuits was well controlled between 170 nm and 880 nm as changing the concentration of the Ag nanoparticle solution (Fig. 19b). In addition, these nanoscale patterned Ag electrodes show good durability for bending and stretching stress on the flexible PDMS substrate.^11^

Fig. 19c shows the nanoscale patterning results from electrohydrodynamic jet printing method with block-copolymer ink.^107^ The microscale pattern formed *via* electrodynamic jetting, and the nanoscale pattern was made by self-assembly of the block-copolymer. The 1 μm scale nozzle was used for the fine control of the jetting. The block-copolymer ink was used for the fabrication of the micro-pattern, and the nanoscale pattern formed at the well-controlled micro-structure. The periodicity of the nanoscale pattern changed from 40 nm to 27 nm *via* molecular weight control of the polymer. The molecular weights (MWs) and composition of the block-copolymers (BCPs) define the size, periodicity and morphology of the patterns.^111^ Increasing of MWs leads to more amphiphilic character and larger size of building block, which induces larger pattern sizes.^118,119^ After patterning with hydrodynamic jet printing, thermal annealing procedure is required for removing residual solvent and activation of self-assembly of BCPs. Thermal annealing generates phase separation of the BCPs into domains oriented perpendicular to the surface with periodicities determined by the MWs of the BCPs. In the case of PS-*b*-PMMA, smaller MWs (25–26k) showed 27 nm of periodical patterns and larger MWs (37–37k) formed 41 nm of patterns size.^111^ In addition, the pattern of the substrate was well controlled based on the self-assembled nanopatterning processes which were composed of the chemical patterning and physical patterning. These results showed various printed features *via* hybrids of the electrohydrodynamic jetting with block-copolymer ink.^111^

**Fig. 20:**
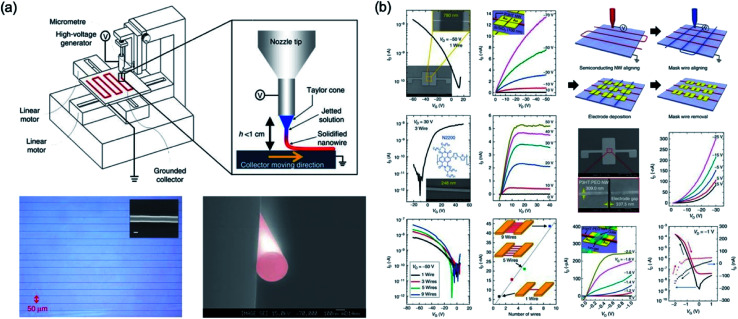
(a) Schematic diagram of ONW printer and NW printing process and optical and SEM image of well-aligned PVK NWs. (b) Electrical characteristics of FET based on ONW and schematic of device fabrication procedures. These figures have been adapted from ref. 76, with permission from the Nature Publishing Group.

Min *et al.*^76^ studied an electrohydrodynamic organic nanowire printer which was controlled individually and aligned at a specific substrate position, as shown in Fig. 20. Their home-made printers consist of an *x*–*y* stage with a linear motor to place the organic nanowire in the desired position, micrometer to control the tip to collector distance, nozzle of about 100 μm, syringe pump, and high voltage generator. To form the organic nanowire, an electrostatic force is applied to the nozzle with viscous polymer solution, and then the viscous polymer solution is injected into the nozzle by a high voltage applied to the nozzle. This results can be applied to electronics such as transistors and inverter circuits by using N/P-type organic nanoscale wires aligned at a desired position.

## 5. Dip pen nanolithography for macro-to-nanometer patterning

Dip pen nanolithography (DPN) was derived from techniques of scanning probe lithography (SPL). SPL techniques were sorted as physical patterning processes that were constructive or destructive. However, DPN patterning was not a direct contact process between tip and substrate. DPN directly delivers materials to substrate from the tip as a likely fountain pen. Basically, a meniscus formed between the scanning tip and the substrate, which serves as a pathway for ink transport. After Mirkin's report,^120^ DPN processes were actively researched by many researchers as next-generation micro-to-nanoscale patterning processes.^121^

**Fig. 21:**
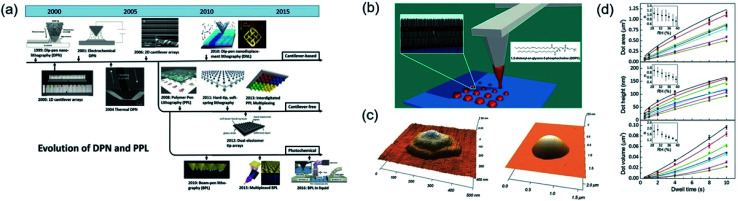
(a) Development roadmap of dip-pen nanolithography. (b) Schematic illustration of dip-pen nanolithography. (c) AFM image of single-dot with dip-pen nanolithography. (d) AFM area, height, and volume of DOPC dot with various dwell times. These figures have been adapted from ref. 121, with permission from the Wiley-VCH and ref. 127, with permission from the Royal Society of Chemistry.

Fig. 21a showed the development roadmap of various DPN technologies.^121^ DPN patterning procedures composed of the cantilever and cantilever-free processes. The tip-attached cantilever was a well-known component for DPN patterning procedures. The atomic force microscope (AFM) tip was used to directly pattern of alkanethiols onto a gold substrate, which was the first invention of DPN.^120^ After this, electrochemical dip-pen nanolithography (E-DPN) procedure suggested for directly patterning of metal and semiconductor materials.^122^ Tip of E-DPN procedure was used not only ink-transport but also electrochemical reaction to convert precursors to metals or semiconducting materials on a substrate. Thermal DPN (tDPN) was developed, which used a heated tip to melt and deposit of solid-state organic inks on a substrate.^123^ The tDPN process allowed one to pattern various solid-state materials with an appropriate melting temperature of each material without solvent for dissolving of solid-state materials. In 2006, parallel DPN (p-DPN) processes reported by Salaita *et al.*^124^ introduced for large area patterning, benefiting from the 55 000-tip array over one square centimeter.^124^ The highly-integrated tip array performed a high-throughput DPN process, which was useful to fabricate various sizes, spacing, and shape with high-speed, rather than single tip based DPN. Cantilever-free DPN procedures were developed for large-area patterning in a cost-effective manner. Polymer pen lithography (PPL), which tip made for silicone rubber known as PDMS, was developed from Huo *et al.*^125^ PPL array had 11 million pyramid-shaped tips obtained from the simple molding process. PPL process had advantages of DPN with micro-contact printing. However, in a high-resolution, the PPL process had limitations caused by their soft-tips. Hard-tip, soft-printing lithography (HSL) was introduced for the realization of ultra-high-resolution printing with cantilever-free media.^126^ HSL had ultra-sharp and hard Si tip arrays on an elastomer layer, which elastomer layer acts as deformable spring to support incompressible tips. HSL had 22 nm tip apex, which performed under 50 nm scale pattern size.

Fig. 21b depicts a schematic illustration of the DPN process with cantilever for patterning of 1,2-dioleoyl-*sn-glycero*-3-phosphocholine (DOPC) molecules.^127^ Patterns of lipid, which is a biomolecule that is soluble in nonpolar solvents, formed with the various scale through transporting lipid ink between cantilever tip and substrate. Fig. 21c shows the AFM image of DOPC single dot patterns, which were controlled from 300 nm to 1 μm *via* simple dwell time control. Short dwell time leads to small size patterns but slightly non-uniform shape and longer dwell time caused a larger pattern size with more uniform shape. Fig. 21d shows the dwell time related to a dot area, height, and volume. With simple dwell time control, each parameter well controlled from micro-to-nanoscale. Almost other patterning procedures could not precisely control their volume of pattern, but this DPN procedure can create exact controllability of area, height, and volume.

## 6. Conclusions

In this review, we have systematically introduced various technologies for micro to nanometer patterning of SBMs, and discussed electronic and optoelectronic applications fabricated by various methods of pattering of SBMs. Nanopatterning with modified optical lithography, NIT, transfer printing, capillary bridge pattering with direct patterning, and inkjet printing techniques were introduced to fabricate nanopatterns of SBMs. The tremendous progress for micro to nanometer patterning of SBMs achieved in various new emerging technologies with the evolution of traditional technologies, as shown in this paper. Through these various efforts, nanopatterned SBMs integrated into electronics and optoelectronics have a brighter future.

## Conflicts of interest

There are no conflicts to declare.

## Supplementary Material
